# Upregulation of neuropeptide Y in cardiac sympathetic nerves induces stress (Takotsubo) cardiomyopathy

**DOI:** 10.3389/fnins.2022.1013712

**Published:** 2022-11-03

**Authors:** Takahide Arai, Hideaki Kanazawa, Kensuke Kimura, Masahito Munakata, Hiroyuki Yamakawa, Ken Shinmura, Shinsuke Yuasa, Motoaki Sano, Keiichi Fukuda

**Affiliations:** ^1^Division of Cardiology, Department of Internal Medicine, Keio University School of Medicine, Tokyo, Japan; ^2^International Medical Center, Department of Cardiology, Saitama Medical University, Saitama, Japan; ^3^Department of Internal Medicine, Kimura Clinic, Kanagawa, Japan; ^4^Department of General Internal Medicine, Hyogo College School of Medicine, Nishinomiya, Japan

**Keywords:** neuropeptide Y, cardiac sympathetic nervous, stellate ganglion, stress, Takotsubo cardiomyopathy

## Abstract

Substantial emotional or physical stress may lead to an imbalance in the brain, resulting in stress cardiomyopathy (SC) and transient left ventricular (LV) apical ballooning. Even though these conditions are severe, their precise underlying mechanisms remain unclear. Appropriate animal models are needed to elucidate the precise mechanisms. In this study, we established a new animal model of epilepsy-induced SC. The SC model showed an increased expression of the acute phase reaction protein, c-Fos, in the paraventricular hypothalamic nucleus (PVN), which is the sympathetic nerve center of the brain. Furthermore, we observed a significant upregulation of neuropeptide Y (NPY) expression in the left stellate ganglion (SG) and cardiac sympathetic nerves. NPY showed neither positive nor negative inotropic and chronotropic effects. On the contrary, NPY could interrupt β-adrenergic signaling in cardiomyocytes when exposure to NPY precedes exposure to noradrenaline. Moreover, its elimination in the left SG *via* siRNA treatment tended to reduce the incidence of SC. Thus, our results indicated that upstream sympathetic activation induced significant upregulation of NPY in the left SG and cardiac sympathetic nerves, resulting in cardiac dysfunctions like SC.

## Introduction

Stress cardiomyopathy (SC), which is also known as Takotsubo cardiomyopathy, is a relatively novel syndrome in cardiology that predominantly occurs in postmenopausal women following emotional or physical stress. The symptoms include chest pain or dyspnea accompanied by electrocardiographic changes, such as ST-segment elevation and T-wave inversion, minimal elevation of cardiac enzyme levels, and transient wall-motion abnormalities. Although the clinical manifestations of SC mimic acute coronary syndrome, coronary angiography has revealed no remarkable coronary stenosis or thrombi in SC. Left ventriculography has also revealed wall-motion abnormalities in the left ventricular (LV) apical segment in SC. However, these abnormalities recover within several days. Elevated catecholamine levels have also been observed in SC, and it has been suggested that a hyperadrenergic response to stress may be the underlying mechanism of pathogenesis of this disease (Maron et al., 2006; Anderson et al., 2007; Prasad et al., 2008; Akashi et al., 2010; Hurst et al., 2010; Nef et al., 2010).

Neuropeptide Y (NPY), which is a 36-amino acid peptide that is co-localized with noradrenaline (NA) in sympathetic nerves innervating the cardiovascular system, is involved in the regulation of cardiovascular and neuroendocrine function (Callanan et al., 2007; Herring, 2015), and mental stress, such as panic disorder (Esler et al., 2004), enhances its release from the cardiac sympathetic nerves. Some previous studies have revealed that NPY may play an important role in the pathogenesis of SC (Wittstein et al., 2005; Szardien et al., 2011).

Presently, suitable animal models are needed to elucidate the precise mechanism of the pathogenesis of SC. In this regard, it has been reported that epilepsy triggers SC in humans (Sakuragi et al., 2007; Stöllberger et al., 2009). Therefore, in this study, we established a new animal model of epilepsy-induced SC and demonstrated how NPY affects cardiac dysfunction in the SC model.

## Materials and methods

### Animal experiments

Nine-week-old Wistar male rats, 12-week-old C57BL/6J male mice and neonatal Wistar rats were purchased from CLEA (Tokyo, Japan). All the experimental procedures and protocols employed in this study were approved by the Animal Care and Use Committee of Keio University.

### Animal model of status epilepticus

Status epilepticus was induced using pilocarpine as previously described (Unsain et al., 2008). Briefly, the 9-week-old Wistar male rats (*n* = 9) were first administered scopolamine methyl bromide (1 mg/kg; ICN Biomedical Inc., Asse-Relegem, Belgium) *via* intraperitoneal injection and after 30 min, they were injected with pilocarpine hydrochloride (380 mg/kg, i.p.; Sigma-Aldrich, St Louis, MO, USA). The seizures were classified according to the Racine Score (RC): 0, no-response; 1, myotonic jerks without rearing; 2, myoclonic jerks with rearing; 3, unilateral forelimb clonus; 4, rearing with bilateral forelimb clonus; and 5, generalized tonic-clonic seizure (Al-Rafiah and Mehdar, 2021). If the RC score reached stage 5, we decided that epilepsy had been induced. Two hours after the onset of status epilepticus, the animals were then anesthetized with pentobarbital to stop seizure activity so that further experiments could be performed.

### Determination of catecholamine and neuropeptide Y levels

Serum catecholamine levels were determined *via* HPLC, while the cardiomyocyte content of NPY in SC and control rats were determined using an ELISA kit for rats (Neuropeptide Y Enzyme Immunoassay Kit; Raybiotech, Norcross, GA, USA). Tissue samples from the left ventricle were homogenized within 30 s in 0.1 N HCl containing 0.1% sodium pyrosulfite as described previously (Kimura et al., 2010). Homogenates were centrifuged (10,000 × *g* for 30 min) and supernatants were collected and assayed.

### Retrograde labeling of sympathetic nerve fibers

To label the central region of cardiac sympathetic nerves, especially at the apex, pseudorabies virus (PRV) was injected into the apex of the LV using a Hamilton micropipette in Wistar rats anesthetized with 1–2% inhaled isoflurane (Vevo Inhalation System; Visual Sonics, Toronto, ON, Canada). Rats were killed 48 h after PRV injection as described previously (Aston-Jones and Card, 2000) and perfused with phosphate-buffered saline (PBS) followed by 4% paraformaldehyde prior to immunofluorescence staining, as described above.

### mRNA analysis

RNA extraction and quantitative reverse transcription-polymerase chain reaction (qRT-PCR) was performed as previously described (Ieda et al., 2004) using the ABI Prism 7500 Sequence Detection System (Applied Biosystems, Foster City, CA, USA). All the samples were analyzed in triplicate. The primers and TaqMan probes for tyrosine hydroxylase (Rn00562500_m1), choline acetyltransferase (Rn01453446_m1), vesicular acetylcholine transporter (Rn00581454_s1), choline transporter (Rn00506029_m1), and NPY (Rn01410145_m1), were purchased from Applied Biosystems. Relative amounts of all mRNA were calculated using the comparative Ct method as described previously. ΔCt is the difference in the Ct values derived from the experimental samples and the glyceraldehyde-3-phosphate dehydrogenase (GAPDH) control.

### Immunohistochemistry staining

The rats were anesthetized using pentobarbital and perfused transcardially with PBS followed by 4% paraformaldehyde. After perfusion, the brains and hearts of the animals were excised, fixed overnight, and cryoprotected in 30% sucrose at 4°C for 7 days before embedding in the optimal cutting temperature (OCT) compound and freezing in liquid nitrogen. Next, cryostat sections were stained with antibodies against Rb132 (a gift from L.W. Enquist, Princeton University, Princeton, NJ, USA) to detect PRV-positive sympathetic nerves, and c-fos (sc-52; Santa Cruz Biotechnology, Santa Cruz, CA, USA) to detect nerve activity, TH (AB152; Chemicon, Temecula, CA, USA) to detect sympathetic nerves, NPY (ab10980; Abcam, Cambridge, UK) to detect NPY-positive sympathetic nerves, and choline acetyltransferase (AB144; Chemicon) to detect parasympathetic nerves. Thereafter, the sections were incubated with secondary antibodies conjugated with Alexa Fluor 488, Alexa Fluor 546, Alexa Fluor 633 (Invitrogen, Carlsbad, CA, USA), and tetramethyl rhodamine isothiocyanate (DAKO, Carpinteria, CA, USA), and nuclei were counterstained with 4′,6′-diamidino-2-phenylindole. Finally, all confocal microscopy observations were performed using an LSM 510 META Confocal Microscope (Carl Zeiss, Oberkochen, Germany). In some of the experiments, paraffin-embedded sections were treated with 10 mM citrate buffer (pH 6) and 20 mM Tris–HCl buffer (pH 9) for antigen retrieval, and signals were visualized using a TSA Direct Kit (Perkin Elmer, Waltham, MA, USA). To quantify the expression level of TH, NPY, and choline acetyltransferase, nerve density was determined using ImageJ software^1^ as described previously (Kanazawa et al., 2010). After that, the percentage of immunopositive neurons was calculated.

### Electrocardiography and echocardiography

To perform electrocardiography and transthoracic echocardiography, the rats were anesthetized with 1–2% isoflurane *via* inhalation. Thereafter, electrocardiography was performed using a Nihon Koden scanner (Tokyo, Japan) with microneedles, while transthoracic echocardiography was performed using a Vevo 770 scanner (Probe: RMV716; Vevo770; Visual Sonics, Toronto, ON, Canada) with 17.5- and 30-MHz probes. Images were acquired using ECG-gated kilohertz visualization (EKV) imaging mode.

### Left ventriculography

A 0.58-mm polyethylene tube was inserted from the right internal cervical artery into the LV in pentobarbital-anesthetized rats. Tube placement in the LV was confirmed by performing pressure checks. Thereafter, contrast medium was injected *via* the tube and left ventriculography was performed using a HITEx system (HITEx, Osaka, Japan).

### Stellate ganglion injections

The rats were anesthetized with 1–2% isoflurane *via* inhalation. Thereafter (mechanically ventilated), their left stellate ganglia (SG) were microinjected with *NPY* siRNA (100 ng/0.5 μl; Santa Cruz Biochemical, Santa Cruz, CA, USA) using a Hamilton micropipette. After injection, transthoracic echocardiography was performed to assess changes in wall motion in the LV apex.

### Langendorff-perfusion of the heart

Hearts were quickly excised from 12-week-old C57BL/6J male mice (*n* = 4 for each group) and perfused with modified Krebs–Henseleit buffer according to the Langendorff procedure as described previously (Shinmura et al., 2007). Thereafter, heart rate (HR), LV systolic pressure (LVSP), and LV end-diastolic pressure (LVEDP) were recorded. Further, LV developed pressure (LVDP) was calculated by subtracting LVEDP from LVSP and used as an indicator of LV systolic function. After 20 min of initial perfusion for stabilization, two different perfusion protocols were examined to investigate the effect of the interaction between noradrenaline (NA) and NPY on LV function. To this end, (1) hearts were exposed to 10^–7^ M NA for 5 min followed by administration of 10^–7^ M NPY, or (2) exposure to 10^–7^ M NPY for 5 min followed by administration of 10^–7^M NA.

### Cardiomyocyte isolation and measurement of length of contraction

A primary culture of neonatal Wistar rat cardiomyocytes was established as previously described (Ieda et al., 2004). In brief, the cardiomyocytes were incubated in a medium containing serum for 24 h after which the medium was replaced with fresh serum-free medium. Beat frequency and length of contraction were then measured using a non-invasive method based on light microscopic video imaging as previously described (Hayakawa et al., 2012). Images of cardiomyocytes were captured using a high-speed camera and motion vectors were calculated at a high spatiotemporal resolution using a block-matching algorithm.

### Cardiomyocyte isolation and measurement of Ca^2+^ transients

Cardiomyocytes were isolated from the LV of 9-week-old Wistar male rats (*n* = 4 for each group) using enzyme digestion methods, as previously described (Shao et al., 2013) with slight modifications. After isolation, the ventricular cardiomyocytes were loaded with 10 μM Fluo-4 AM (Invitrogen, Carlsbad, CA, USA) to monitor localized changes in intracellular [Ca^2+^]. The experimental chamber was mounted on the stage of an LSM 510 META Confocal Microscope (Carl Zeiss). Subsequently, Fluo-4 was excited using a 488-nm argon laser, while the Ca^2+^ transient frequencies and peak to transients were determined from the line scans.

### Statistical analysis

Data were presented as the mean ± SD. Significant differences between two means were determined by performing two-tailed, unpaired Welch’s *t*-tests. *P* < 0.05 was considered significant. Statistical analyses were performed using IBM SPSS Statistics, Version 21.0 (SPSS, Inc., Chicago, IL, USA).

## Results

### New animal model of epilepsy-induced stress cardiomyopathy

**FIGURE 1:**
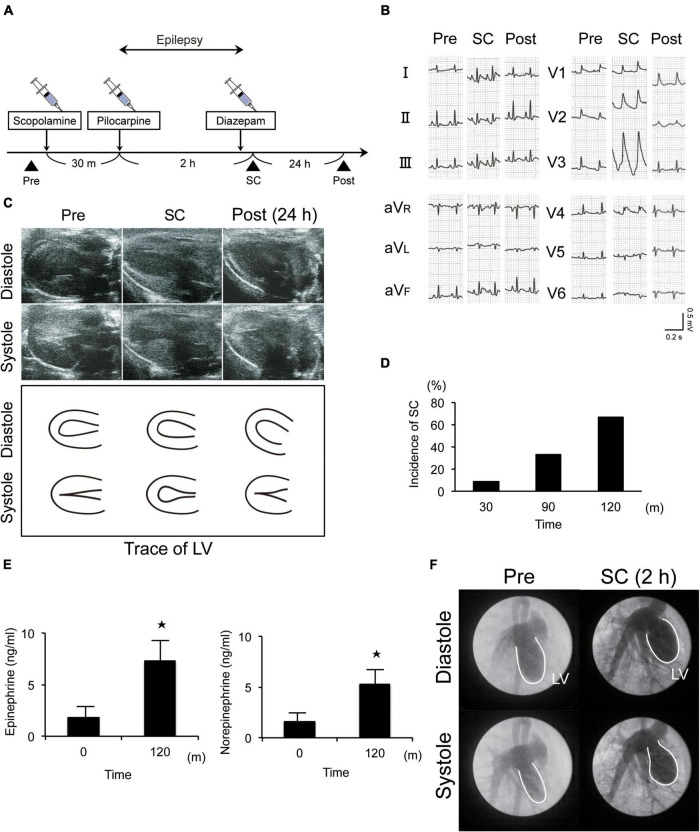
New animal model of epilepsy-induced stress cardiomyopathy (SC). **(A)** Protocol for the induction of SC in Wistar rats using pilocarpine. **(B)** Electrocardiograms revealing typical ST elevation during SC. Pre, pre-inducing epilepsy; Post, post-inducing epilepsy; **(C,F)** Echocardiography (**c**) and left ventriculography **(F)** revealed that wall motion of the left ventricle (LV) apical segment was akinetic or dyskinetic during SC. **(D)** Incidence of epilepsy-induced SC (*n* = 9). **(E)** Upregulated serum epinephrine, norepinephrine, and dopamine levels 2 h after the induction of epilepsy (*n* = 4). Where appropriate, data are provided as the mean ± SD. *P < 0.05 compared with 0 min **(E)**.

Epilepsy was induced in Wistar rats *via* the injection of scopolamine, followed by pilocarpine, a muscarinic receptor agonist (Figure 1A). Electrocardiography and echocardiography revealed typical ST elevation (Figure 1B) and akinesia/dyskinesia of the LV apical segment, respectively. Further, wall motion in the LV apical segment completely recovered by the following day (Figure 1C and Supplementary Movies 1–3). These changes occurred in 11% (1/9), 33% (3/9), and 67% (6/9) of the rats within 30 min, 1 h, and 2 h, respectively, after the induction of epilepsy (Figure 1D). Furthermore, the rats also showed approximately threefold increases in serum epinephrine and norepinephrine levels at 2 h (Figure 1E). Left ventriculography also revealed akinesia or dyskinesia of the LV apical segment (Figure 1F and Supplementary Movies 4, 5). Thus, taken together, our established model mimicked the clinical features of SC.

### Pseudorabies virus staining of the central portions of sympathetic nerve fibers and c-FOS expression

**FIGURE 2:**
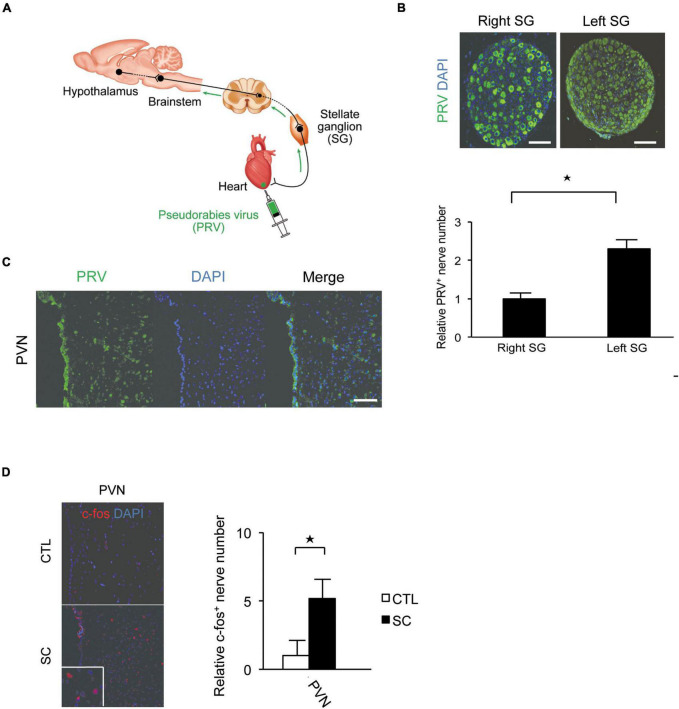
Pseudorabies virus (PRV) staining of the central portions of sympathetic nerve fibers innervating the apex of the left ventricle. **(A)** Schema of retrograde labeling by PRV. SG, stellate ganglion. **(B)** Neurons were labeled by PRV. More neurons were labeled by PRV in the left SG than in the right SG. DAPI, 4′,6′-diamidino-2-phenylindole. **(C)** PRV staining revealed that the central portion of the cardiac sympathetic nerve originated mainly in the paraventricular hypothalamic nucleus (PVN). Scale bars: 50 μm **(B)**; 100 μm **(C)**. **(D)** Immunofluorescence staining for c-fos in the PVN of the control and SC groups. Quantitative analysis of c-fos-positive cells is shown (*n* = 5). *p < 0.05.

Pseudorabies virus staining revealed that the central portion of the cardiac sympathetic nerves exists in the paraventricular hypothalamic nucleus (PVN), the main central site for the integration of sympathetic nerve activity and regulation of cardiovascular function. Furthermore, more neurons were labeled by PRV in the left SG than in the right SG (Figures 2A–C).

We also confirmed that the SC model showed an increased expression of the acute phase reaction protein, c-fos, in the PVN (Figure 2D).

### Significant upregulation of neuropeptide Y expression in the left stellate ganglion and cardiac sympathetic nerves

**FIGURE 3:**
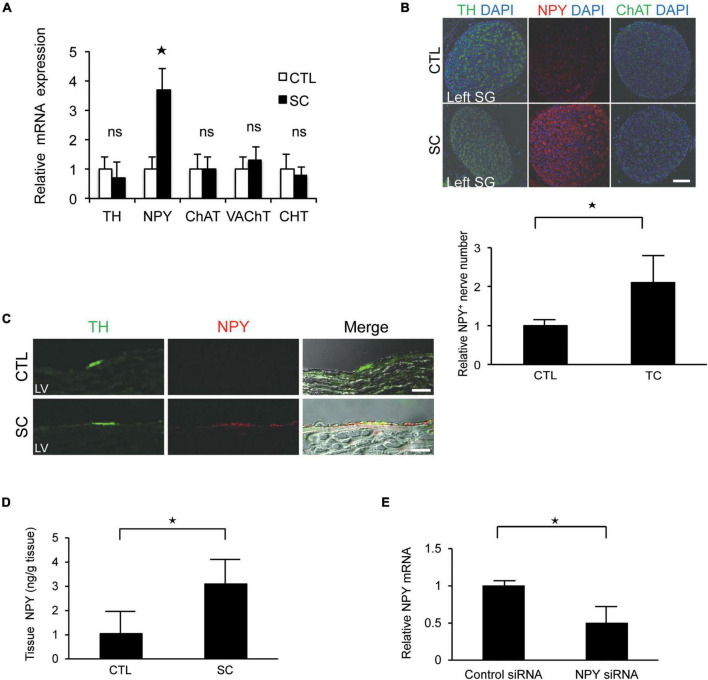
Upregulation of neuropeptide Y (NPY) expression in the left stellate ganglion (SG) and cardiac sympathetic nerves. **(A)** mRNA expression of various neuronal markers in the left SG in the control (CTL) and stress cardiomyopathy (SC) groups. SGs in SC model were collected 2 h after the commencement of epilepsy. NPY expression was significantly increased in the SC model, whereas there was no increase in the expression of the choline transporter (CHT), choline acetyltransferase (ChAT), vesicular acetylcholine transporter (VAchT), or tyrosine hydroxylase (TH; *n* = 5). **(B,C)** Triple-immunohistochemistry staining of the left SG and left ventricle (LV) for TH (green), NPY (red), and 4′,6′-diamidino-2-phenylindole (DAPI; nuclei; blue). NPY upregulation was observed in the SG and cardiac sympathetic nerve of the LV in the SC model. Scale bars: 50 μm **(B)** and 100 μm **(C)**. **(D)** NPY content of the apical part of the LV in control and SC rats (*n* = 6). **(E)** Expression level of *NPY* mRNA significantly reduced following the injection of *NPY* siRNA into left SG compared to control siRNA (*n* = 6). Where appropriate, data are provided as the mean ± SD. *P < 0.05 relative to the control.

There were no significant differences between the control and SC animals with respect to the expression levels of tyrosine hydroxylase (TH; a marker of sympathetic nerves), choline transporter, choline acetyltransferase, or vesicular acetylcholine transporter (markers of cholinergic nerves). However, the SC model showed significantly increased NPY expression (Figure 3A). Further, immunohistochemistry staining revealed that NPY was highly expressed in the SG and cardiac sympathetic nerves in the LV only in the SC model (Figures 3B,C). ELISA also revealed marked increases in NPY levels in the LV apex in the SC model (Figure 3D).

To confirm the importance of NPY, *NPY* siRNA treatment was performed. Thus, we observed that the expression level of *NPY* mRNA was significantly reduced when *NPY* siRNA was injected into the left SG compared with the control (Figure 3E). Additionally, the incidence of SC tended to decrease to 0% (0/4) after *NPY* siRNA injection into the left SG.

### Left ventricular dysfunction induced by increased neuropeptide Y expression

**FIGURE 4:**
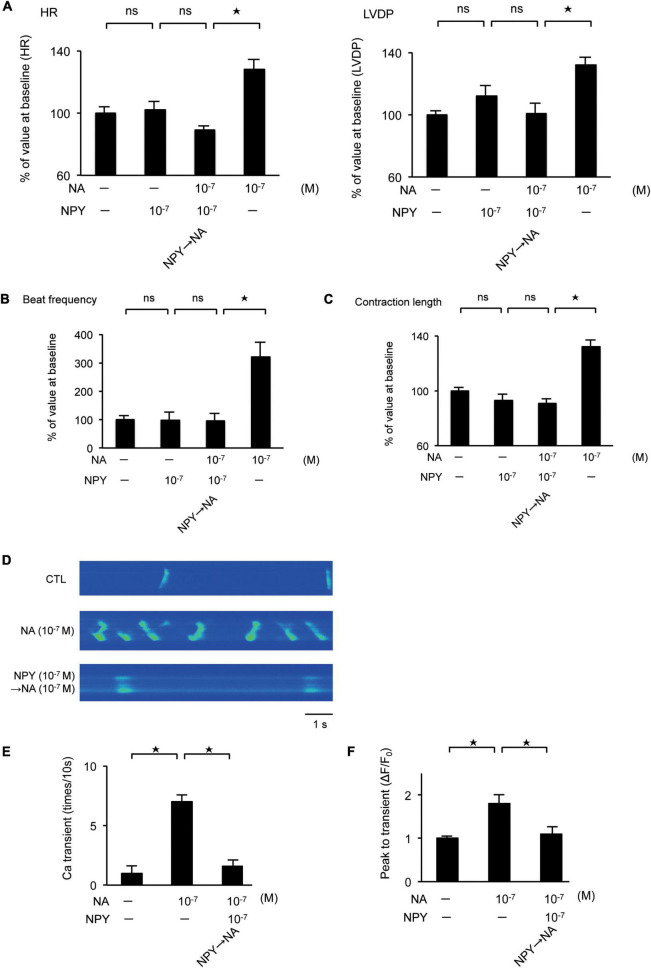
**(A)** Investigation of the effects of neuropeptide Y (NPY) on left ventricle (LV) contraction using the Langendorff procedure. Pretreatment with NPY completely blocked NA-induced increase in heart rate and LV developed pressure (*n* = 4 for each group). **(B,C)** The measurement of beat frequency and contraction length using primary cultured cardiomyocytes revealed that when hearts were exposed noradrenaline (NA), beat frequency and contraction length significantly increased (*n* = 4). Conversely, when hearts were first exposed NPY, beat frequency and contraction length did not increase after NA administration (*n* = 4). **(D–F)** Effects of NPY on ventricular cardiomyocytes isolated from adult rats based on the measurement of the frequency of Ca^2+^ transients and the peak to transients. The frequency of Ca^2+^ transients and the peak to transients in isolated ventricular cardiomyocytes increased significantly when cells were exposed to NA only, but increases were not observed when cells were first exposed to NPY and then administered with NA (*n* = 4 for each group). *p < 0.05, ns: not significant.

We investigated the effect of the interaction between NA and NPY on LV function using Langendorff-perfused mouse hearts. NA exhibited a positive inotropic and chronotropic effect, while NPY showed neither positive nor negative inotropic and chronotropic effects. When the hearts were first exposed to NPY, further NA administration failed to increase HR and LVDP (Figure 4A). Using primary cultured rat neonatal cardiomyocytes, we observed that NA-induced increases in beat frequency and contraction length were completely abrogated by NPY pre-treatment (Figures 4B,C). These findings indicated that NPY could interrupt β-adrenergic signaling in cardiomyocytes when exposure to NPY precedes exposure to NA.

Furthermore, we investigated the effects of NPY on ventricular myocytes isolated from adult rats by measuring the frequency of Ca^2+^ transients as well as the peak to transients using Fluo-4 (Shah et al., 2010). Thus, we observed that the frequency of Ca^2+^ transients and the peak to transients in isolated ventricular myocytes increased significantly when the cells were exposed to NA only. Conversely, increases were not observed when cells were first exposed to NPY before NA (Figures 4D–F).

## Discussion

In this study, we demonstrated that upstream sympathetic activation could induce significant upregulation of NPY in left SG and cardiac sympathetic nerves, resulting in cardiac dysfunction like SC by using a new animal model of epilepsy-induced SC.

Given that the precise molecular mechanisms underlying the pathogenesis of SC remain unclear, suitable animal models are needed to provide clarifications in this regard. Reportedly, epilepsy triggers SC in humans (Sakuragi et al., 2007; Stöllberger et al., 2009). Therefore, in this study, the epilepsy-induced SC model was established, and we observed that it sufficiently mimicked the clinical features of SC, such as regional LV dysfunction with complete recovery, ECG changes, no coronary obstruction, and elevated serum catecholamine levels. There are some reports regarding animal models of SC. Specifically, a model established by injecting model animals with β-adrenergic agonist isoprenaline has been proposed (Shao et al., 2013); however, evaluating the factors apart from catecholamine seems to be challenging. Reportedly, a rat SC model can be established *via* immobilization-induced stress (Ueyama et al., 2002); however, taking advantage of genetic techniques will require an animal model establishment strategy that can be applied in both mice and rats. The epilepsy-induced SC model established in this study may also be applied to mice.

Pseudorabies virus is a retrograde neuronal tracer (Standish et al., 1994; Jansen et al., 1995). We injected PRV into the LV apex to detect the central region of cardiac sympathetic nerves, especially at the apex. PRV staining revealed that the central portion of the cardiac sympathetic nerves exists in the PVN. PVN was reported to be the main central site for the integration of sympathetic nerve activity and regulation of cardiovascular function (DiMicco et al., 2002; Kenney et al., 2003; Ueyama et al., 2006; Infanger et al., 2010). We next confirmed that the SC model showed an increased expression of the acute phase reaction protein, c-fos, in the PVN. c-fos is a type of acute phase reaction protein that is known as a marker of functional activation of the brain (Martinez et al., 2002). This means the central portion of the cardiac sympathetic system was activated in the SC model, which could lead to the subsequent change to the peripheral sympathetic nervous systems.

More neurons were labeled by PRV in the left SG than in the right SG. Thus, left SG seems to be more important with regard to the influence of the LV apex. Reportedly, in severe heart failure, the cardiac sympathetic nerve shows cholinergic neurotransmitter switching (Kanazawa et al., 2010) in SG. In this study, we investigated the mRNA expression levels of various neuromarkers in the SG in a rat model of epilepsy-induced SC. No significant differences were observed between the control and SC groups with respect to the expression levels of the markers of sympathetic and cholinergic nerves. Approximately 60% of the SG includes NPY immunoreactive neurons (Masliukov et al., 2012). However, the SC model showed significantly increased NPY expression compared to the baseline expression.

The majority of NPY immunoreactive fibers are post-ganglionic, originating from the SG and colocalizing with tyrosine hydroxylase (Masliukov et al., 2016). Sympathetic nerve terminals, which can act as local neuromodulators in various cardiovascular disorders, represent one of the main sources of NPY (Tan et al., 2018). NPY is also released in response to sympathetic stimulation and functions as a co-transmitter of NA, and significant co-transmitter release generally occurs only after high-level neural stimulation. For example, in cases of acute myocardial infarction and congestive heart failure, characterized by high-level sympathetic drive, NPY can be released in addition to NA (Herring, 2015). Further, it has been reported that NPY is involved in the pathogenesis of SC as well as vasospasm and ventricular arrhythmia following myocardial infarction (Herring et al., 2019; Kalla et al., 2020). Our results demonstrated that NPY upregulation and release can occur in cardiac sympathetic nerves activated by considerable emotional or physical stress, leading to LV dysfunction.

However, little is known regarding the pathophysiological role of NPY as a co-transmitter in the cardiovascular system. Thus, we investigated its interaction with NA with respect to LV function. Our results demonstrated that NPY did not only show a negative inotropic effect, but also strongly blocked β-adrenergic stimulation. Thus, NPY was co-released with NA in response to stress. Additionally, the increase in the expression level of NPY could be related to the frequency of sympathetic nervous stimulation. Taken together, the spill-over of NA and NPY from pre-synaptic terminations at the LV level can lead to myocardial dysfunction.

There is a report that NPY has a negative inotropic effect on the myocardium from 7-day-old rats. On the other hand, a positive inotropic effect was seen in the myocardium of 21-day-old rats. NPY had little effect on the myocardium of 100-day-old rats. The reaction of the myocardium to NPY appears to differ with age (Zverev et al., 2014). The study examined the reaction of the myocardium after stimulation. In our *in vitro* study, we revealed that NPY has no negative inotropic effect on myocardium from neonate rats. We examined spontaneous myocardium contractions using microscopic video imaging. The condition of this experiment was different from that in the reference, thus our data revealed a different tendency. On the other hand, in the Langendorff procedure, we used 12-week-old mice and revealed that NPY showed neither positive nor negative inotropic and chronotropic effects. This result is in agreement with the findings of Zverev et al. (2014). In general SC occurred in adults and is uncommon in juveniles. Experiments using older animals seem to show similar conditions with SC. The reaction of the myocardium to NPY seems to differ according to age. The reference suggested that one reason for this phenomenon could be the different expression of the NPY receptor. However, the precise mechanism of this was not clarified. Further studies are needed to elucidate this phenomenon.

There remains one important question: why does LV dysfunction only occur in the apical LV? Our data using PRV suggested that nerve fibers from the left SG primarily innervate the apical LV, while those from the right SG innervate a broader distribution of cardiac muscle, including the right ventricle and basal LV. Thus, the effect of NA and NPY under stress conditions in the left SG may be limited to the apical LV. Further studies are required to test this hypothesis.

This study had some limitations. First, we did not perform an experiment involving NPY overexpression in our SC model. Second, the mechanism how hypothalamic activation leads to the upregulation of NPY seems to be unclear in this study. Thus, further studies are necessary.

In conclusion, upstream sympathetic activation could induce significant upregulation of NPY in left SG and cardiac sympathetic nerves, resulting in cardiac dysfunction like SC. Our findings also suggested that NPY may be an attractive target for the treatment of SC.

## Data availability statement

The datasets presented in this study can be found in online repositories. The names of the repository/repositories and accession number(s) can be found in the article/Supplementary material.

## Ethics statement

The animal study was reviewed and approved by Animal Care and Use Committee of Keio University.

## Author contributions

TA designed and performed the experiments, analyzed the data, and wrote the manuscript. HK, KK, SY, and MS contributed to the experimental design and wrote the manuscript. MM and HY performed the experiments. KS contributed to the experiments related to the Langendorff procedure. KF initiated and directed the entire study, designed the experiments, and wrote the manuscript. All authors contributed to the article and approved the submitted version.

## Footnotes

1
http://rsbweb.nih.gov/ij/

## Supplementary material

Supplementary Movie 1 EKV-mode echocardiography before the induction of epilepsy in rats.

Supplementary Movie 2 EKV-mode echocardiography within 2 h of the induction of epilepsy reveals wall motion abnormalities of the left ventricular apical segment in rats.

Supplementary Movie 3 EKV-mode echocardiography reveals complete recovery within 24 h of left ventricular wall motion of the apical segment in rats.

Supplementary Movie 4 Left ventriculography before the induction of epilepsy rats.

Supplementary Movie 5 Left ventriculography within 2 h of the induction of epilepsy reveals wall motion abnormalities of the left ventricular apical segment in rats.
